# *Brucella* Seroprevalence in a High-Risk Population in Greece: A Cross-Sectional Study

**DOI:** 10.1155/2018/8751921

**Published:** 2018-12-25

**Authors:** Panagiotis Andriopoulos, Dimitrios Floros, Natalia Gioti, Anargiros Mariolis, Andrea Paola Rojas Gil, Maria Tsironi

**Affiliations:** ^1^Laboratory of Epidemiology and Prevention of Hemoglobinopathies, Acute and Chronic Diseases, Department of Nursing, University of Peloponnese, Valioti & Plateon, 23100 Sparta, Laconia, Greece; ^2^Areopoli Health Center, Areopoli 23062, Laconia, Greece; ^3^Laboratory of Biology and Biochemistry, Department of Nursing, University of Peloponnese, Valioti & Plateon, 23100 Sparta, Laconia, Greece

## Abstract

**Introduction:**

Brucellosis is a zoonosis with high occupational risk. However, seroprevalence of* Brucella* antibodies among occupational groups is not known, since studies in endemic countries are rare.

**Methods:**

A cross-sectional seroprevalence study was conducted among livestock farmers in an endemic region in Greece. A low-risk group of individuals that just moved in the region was used as controls. Rose Bengal, Wright standard tube agglutination (STA) tests, and specific IgG and IgM antibodies using ELISA were evaluated; differences and odds ratios were calculated. Results were compared with studies from other endemic regions.

**Results:**

100 livestock farmers and family members and 34 first-year students were enrolled. Rose Bengal results were 18% positive versus 0% (p=0.007); Wright STAs for* Brucella melitensis* were 8% versus 2.9% (p=0.448) and for* Brucella abortus* they were 2% versus 2.9% (p=0.588). ELISA IgG was positive in 8% of farmers versus 2.9% of students (p=0.448). Parallel testing with Rose Bengal and ELISA IgG was positive in 3% versus 0% (p=0.571). No significant odds ratios were calculated for Wright STAs and ELISA IgG.

**Conclusions:**

Healthy livestock farmers may present with positive Rose Bengal test but this translates to true seroprevalence in only a small proportion. Livestock farmers have no significant seroprevalence that may obscure diagnosis of acute brucellosis in clinical settings.

## 1. Introduction

Brucellosis remains the most common zoonosis worldwide [1]. Despite the eradication efforts, reports from all over the world reveal the burden of the disease in not only well-known endemic countries [2–5] but also in countries where brucellosis was not a major health problem until recently [6–8]. Brucellosis is a disease with occupational interest, since a great percentage of the affected population is livestock farmers, their families, slaughterhouse workers, and veterinarians [9–12]. Infection in occupational settings occurs through direct contact from cuts and skin abrasions, inhalation of contaminated aerosols, and contact with conjunctival mucosa and of course by consuming contaminated dairy products [13, 14].

Brucellosis has been a major health problem in Greece. Many reports from different parts of the country point to the need for a continuous surveillance system [10, 13–17]. Most of them have identified occupational exposure and residence in rural areas as common risk factors for the development of infection. Diagnosis of brucellosis in these reports is mainly based on isolation of* Brucella* spp. Even though isolation of the pathogen by culture of body fluids is considered the gold standard [18], fast and reliable serological tests provide results rapidly and are also used for diagnosis [19, 20]. Enzyme-linked immunosorbent assay (ELISA) tests are another alternative, both in clinical settings and in prevalence studies. Individual specific IgG and IgM immunoglobulins can be measured rapidly but at a much greater cost than simple agglutination tests [21].

Seroprevalence of* Brucella* antigens has been documented in studies from endemic areas in Asia [22–24], Sub-Saharan Africa [25, 26], and Turkey [27]. Seroprevalence studies from Europe are limited. Reports on serological tests are limited to diagnostic procedures on patients [28] or on follow-up of former patients [19]. Aim of this study is to evaluate the seroprevalence of* Brucella* antigens in a high-risk population of an endemic region in Europe and compare the results with low-risk individuals from the same area.

## 2. Materials and Methods

Laconia is a mostly rural area located in Peloponnese, southern Greece. Brucellosis is a common health problem and has been described elsewhere [10, 13, 15]. Livestock farmers (mainly flocks of sheep and goats) are scattered all over the state. Healthy participants from the official state database were randomly selected. A short questionnaire on possible acute infection during the past 6 months was completed including information on fever, malaise, arthralgias, low back pain, headache, and other common clinical symptoms of brucellosis. Blood samples were obtained in order to perform serology tests. Cluster sampling had been performed and, for every consenting professional, family sampling was sought. In total, 100 different individuals from 57 sampling sites participated in the study. Sampling was performed from October 2015 to September 2016.

In order to compare serology results from high- and low-risk populations, we asked first-year students of our Department of Nursing in Sparta to participate in the study. Students were eligible to take part in the study if they had come from urban areas from other parts of Greece except Peloponnese and had no family occupational history that might have led to contact with* Brucella* spp. and no history of past infection. They also completed the same questionnaire as the livestock farmers. The recruitment was completed within 1 month of residency in Laconia in two parts, newcomers of September 2015 and of September 2016. From all candidates, 34 were randomly selected. We did not include any other lower-risk individuals that had a permanent residency in our state in the control group because we decided to have different population person and place characteristics in terms of descriptive epidemiology.

Serological evaluation was performed using* Brucella* agglutination tests: the Rose Bengal slide agglutination test (RB) and the Wright standard tube agglutination (STA) test with reagents by Linear Chemicals S.L.U. Rose Bengal antigen is a suspension of* Brucella abortus* colored with Rose Bengal stain. For our study, blood sampling was taken by venipuncture, and the serum was separated from each sample after centrifuging. Sera were stored and divided into aliquots at −20°C until use. For RB, 0.05 ml of serum was mixed with an equal volume of antigen on a test plate to produce a zone that is approximately 2 cm in diameter. In ambient temperature after agitation, the mix was observed for agglutination and any visible reaction was considered positive. No dilutions were performed for Rose Bengal test. All sera were routinely tested with STA both in low and in high dilutions (from 1/40 to 1/1600) in order to avoid negative results due to prozone phenomenon. STA was performed by adding 0.05 ml of* abortus* and* melitensis* antigens in low- and high-diluted sera. The dilutions were observed for agglutination after gentle agitation. Agglutination in any dilution was considered as positive result.* Brucella abortus* antibodies were measured by ELISA using MP Biomedicals Germany GmbH's* Brucella* IgM ELISA and* Brucella* IgG ELISA kits. Results higher than 50 U/ml and 40 U/ml accordingly were considered positive.

Statistical analysis of the data was conducted using the SPSS v23 and STATA 14.0 packages. Descriptive statistics (frequencies, means, and 95% confidence intervals (CI)) were measured for each test. Differences in categorical variables were evaluated by Fisher's exact test for small samples. Differences in means were evaluated with Student's* t*-test. In this cross-sectional study, odds ratios (ORs) with 95% confidence intervals (95% CI) and p values were calculated to estimate the probability of positive results in the two groups of participants. Results were considered statistically significant when p < 0.05.

Written informed consent was obtained from all participating individuals. Blood sampling was performed after completing the questionnaires and all safety precautions were undertaken. The study was approved by the University of Peloponnese's ethics committee in accordance with the ethical standards laid down by the 1964 Declaration of Helsinki and its later amendments.

## 3. Results

**Figure 1:**
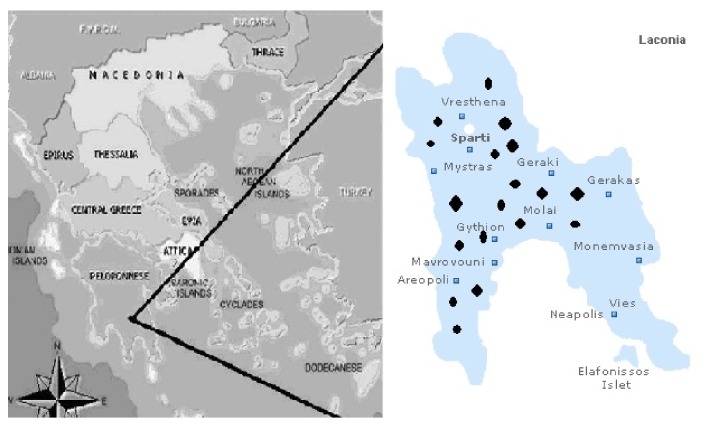
**Map of Laconia and sampling sites. **Black dots represent the various sites of cluster sampling of high-risk populations and the white dot represents the city of Sparta where the students (low-risk population) attend the university.

100 livestock farmers and family members and 34 students of the Nursing Department participated in the study.  Figure 1 depicts the geographical distribution of the 19 sites (villages) where cluster sampling was performed. Serological results are summarized in Table 1. The occupationally exposed high-risk population was, as expected, older than the group of students (mean age 57.7 years (95% CI 54.31-60.97) versus 19.5 years (95% CI 19.23 – 19.58)) and predominantly male. 18% of the high-risk group had positive Rose Bengal test, 8% Wright* melitensis* STA, and 2%* abortus* STA (positive results in Rose Bengal test in all patients with positive STA tests; however, some of them had positive STA for* melitensis* and others tested positive for STA* abortus*). The students had no positive Rose Bengal and only one tested positive for both STA tests. The difference between groups was significant only for Rose Bengal (p=0.007).

ELISA serology tests provided different results. No positive values of the test were recorded in the high-risk group for IgM antibodies (mean IgM for livestock farmers 4.33 U/ml (95% CI 2.37 – 6.28)). Two students had positive IgM antibodies in the absence of positive agglutination tests and this was considered to be random error. No statistically significant differences were recorded in titers of IgG antibodies (mean of livestock farmers 7.96 U/ml (95% CI 3.11 – 12.8) versus 4.06 U/ml (95%CI 0.52 – 7.59)) of students (p=0.367). The difference in positive results was also not significant: 8% in the high risk versus 2.9% in low risk (p=0.448). Only 3% of the livestock farmers had both Rose Bengal and ELISA IgG positive and none of the students had that (p=0.571).

We calculated odds ratios for the tests in order to evaluate the probability of a positive test according to the risk group (Table 2). No significant ORs were found for* abortus* STA,* melitensis* STA, and ELISA IgG antibodies. ORs cannot be calculated for negative results, so no ORs could be obtained for Rose Bengal and ELISA IgM antibodies.

## 4. Discussion

Greece is the country with the highest reported annual incidence of brucellosis in the EU [29]. Lytras et al. [10] in their study on the incidence patterns in the country identified the occupational risk factors associated with the disease. The annual incidence among livestock farmers was 7.1 per 100.000 per year. A brucellosis control program is implemented in the country and Laconia is a state in the vaccination zone, where all reproductive animals are legally required to be inoculated against* Brucella*. Livestock farmers were also identified as high-risk group in other studies from Greece and the Balkan Peninsula [13, 15, 16, 30]. Cluster random sampling in the families of livestock farmers was selected because usually all the members (mostly the male ones) are predominantly or occasionally in close contact with the animals [10, 16].

Serological agglutination tests are used for rapid diagnosis of brucellosis worldwide. Several studies have evaluated their sensitivity and specificity on patients and healthy individuals [20, 31, 32] with various results but, as shown elsewhere [19], these results have to take into account the prevalence of the disease in order to provide accurate information. Diaz et al. [20] have suggested titration and dilution for the RB test but this procedure is not regularly reported in the literature. Wright STA tests are considered positive in dilutions equal to or greater than 1/160; some researchers even suggest 1/320 in endemic areas. In a large case series of acute brucellosis [13], we found that no such threshold is safe, since many patients had culture-proven brucellosis with positive results only in 1/80 dilutions. In our study, we documented a difference between STAs for* abortus* and* melitensis* in livestock farmers. This might be explained by the predominance of sheep and goats in the flocks, but this is only a hypothesis.

IgM and IgG antibodies have been used for diagnosis of brucellosis for decades; however, commercial kits in regular practice have been widely available in the past decade. Various studies have evaluated their performance [31–33, 36]. Most studies focus on the reliability of the essays to diagnose chronic and relapsing cases. In acute brucellosis, the results are usually the same with serology. However, in developing countries and rural areas where the disease is highly endemic, ELISA antibodies are rarely used on seroprevalence studies and the prevalence of IgG antibodies among high-risk populations is not regularly reported. In our study, the positive RB test was not confirmed by ELISA and the probability of having a positive result of IgG antibodies in livestock farmers as calculated by ORs was also not significantly higher. This difference between serology and ELISA confirms a previous report that estimated the positive prognostic value of serology tests for brucellosis to only 11.4% [19]. Two of the low-risk individuals (5.9%) had a positive IgM ELISA test without any symptoms or signs of the disease. This is a confirmation of a well-discussed problem in brucellosis that no laboratory result can provide diagnosis in the absence of clinical and epidemiological data that point to the disease [5, 20, 28].

A MEDLINE/Google Scholar search was performed using keywords* Brucella*/brucellosis and seroprevalence, brucellosis seroprevalence, and high risk occupation. The majority of the studies focused on seroprevalence as a diagnostic procedure in order to identify patients. The aim of our study was to measure seroprevalence of brucellosis in healthy individuals, so we compared our results only with studies that had such information. Moreover, we selected reports from livestock farmers and not veterinarians or abattoir workers, since the exposure risk is not the same. In total, we found only 11 studies that fulfilled our criteria. In Table 3, the results from our study are compared with other relevant reports from endemic areas. In our study, 18% of the high-risk population had positive RB and 8% positive IgG antibodies and Wright STA was positive in 2% for* abortus* and 8% for* melitensis* antigens. Studies from different areas, the Mediterranean Basin [27, 31, 33], sub-Saharan Africa [12, 25, 26], the Middle East [34, 35], and Asia [22–24], provide a different serological profile in high-risk populations and in healthy individuals where available. The tests used to measure seroprevalence differ in each study, but overall positive RB ranges from 2% to 18.6% and ELISA IgG from 2.86% to 16.7% (either ELISA IgG or ELISA IgG and agglutination tests). 7 out of 11 studies reported results only from agglutination tests.

Several limitations are present in our study. First, livestock farmers from only one region of Greece were studied and the disease is endemic in the whole country. Second, implications can be made for only one high-risk occupational group and not veterinarians or abattoir workers. Finally, dilutions in the Rose Bengal test were not performed and this might explain the relatively high positive results in this test.

## 5. Conclusions

In conclusion, we performed a seroprevalence study of brucellosis in a high-risk occupational group and compared it with a low-risk population. We found minimal differences for positive results in Wright STA tests and in ELISA IgG antibodies and a significant difference in Rose Bengal test, a difference that was minimized when RB and ELISA IgG were combined. To our knowledge, this is the only seroprevalence study of brucellosis in healthy individuals in an endemic area from Europe and it points to the fact that livestock farmers have no significant seroprevalence that may obscure diagnosis of acute brucellosis in clinical settings.

## Figures and Tables

**Table 1:** Serology results in high- and low-risk populations.

	**Livestock farmers**	**Students**	***p value***
**Age**	57.7 (54.31 – 60.97)	19.5 (19.52 – 19.58)	***<0.001***
**Sex**	51% male	5.9% male	***<0.001***
**Rose Bengal Test**	18% (18/100)	0% (0/34)	***0.007***
**Wright *abortus* STA**	2% (2/100)	2.9% (1/34)	0.588
**Wright *melitensis* STA**	8% (8/100)	2.9% (1/34)	0.448
**ELISA *abortus* IgM**			
Mean	4.33 U/ml (2.37 – 6.28)	20.23 (13.78 – 26.70)	***<0.001***
Positive	0% (0/100)	5.9% (2/34)^1^	0.063
**ELISA *abortus* IgG**			
Mean	7.96 U/ml (3.11 – 12.8)	4.06 U/ml (0.52 – 7.59)	0.367
Positive	8% (8/100)	2.9% (1/34)^1^	0.448
**Rose Bengal and ELISA IgG positive**	3% (3/100)	0% (0/34)	0.571

Data are presented as mean (95% CI) for continuous variables and as % percentages (frequencies) of positive results in categorical. STA tests were positive at 1/80 dilutions. Some of the livestock farmers with positive RB test had positive STA tests (most for *melitensis* and the rest for *abortus*). ELISA IgG tests were considered positive at values >40 U/ml and IgM at >50 U/ml.

^1^These results were considered random error due to lack of any symptoms of disease on the day of blood sampling and on a follow-up 2 weeks later.

**Table 2:** Odds ratio for positive test in high- versus low-risk population.

	**Odds ratio**	**95% confidence interval**	***P value***
**Rose Bengal**	-		
**Wright *abortus***	0.67	(0.05 – 7.74)	*0.75*
**Wright *melitensis***	2.87	(0.34 – 24.21)	*0.31*
**ELISA IgM **	-		
**ELISA IgG**	2.87	(0.34 – 24.21)	*0.31*

Odds ratios were calculated if possible. In ratios with no positive results, no ORs could be calculated.

**Table 3:** Comparative results of seroprevalence studies in endemic areas.

	**RB**	**Wright STA**	**ELISA G**	**ELISA M**	**Parallel testing**	***Study***
**Mediterranean**						
Greece (100)	18%	8% (M)	8%	0%	3%^1^	
Turkey (573)	11.9%	5.4% (N/A)				*Vancelik et al [27]*
Spain (90)^2^	0%	0% (A)	0%	0%		*Gomez et al [28]*
Turkey (528)	4%	5.2% (N/A)				*Kose et al. [33]*
**Africa**						
Angola (132)					16.7%^3^	*Mufinda et al. [12]*
Tanzania (67)	2%					*Swai et. al. [25]*
Uganda (140)	18.6%					*Tumwine et al.[26]*
**Middle East**						
Iran (292)		5.5 % (N/A)				*NIkokar et al [34]*
Iran (250)^4^		6.4% (N/A)				*Esmaeli et al.[35]*
**Asia**						
Mongolia (2856)	11.1%					*Tsend et al. [22]*
India (121)^5^	9.91%	9.09% (N/A)	16.52%			*Sharma et al. [23]*
Bangladesh (386)					2.86%^6^	*Rahman et al [24]*

Results from seroprevalence studies in livestock farmers. In each study, the number of subjects is mentioned after the country. The study from Spain (Gomez et al.) is included because it provides the only other available seroprevalence data from Europe we identified in our literature search. In STA tests, M denotes *melitensis*, A denotes *abortus*, and N/A denotes being not mentioned.

^1^RB and ELISA IgG.

^2^Healthy blood donors.

^3^Positive STA and ELISA.

^4  5^All high-risk groups.

^6^Positive RB, STA, and ELISA.

## Data Availability

The SPSS and STATA files with the data used to support the findings of this study are available from the corresponding author.
